# Assessment of Volumetric Changes Using Megavoltage-Cone Beam Computed Tomography (MV-CBCT) for Adaptive Radiotherapy in Head and Neck Cancer: A Case Series

**DOI:** 10.7759/cureus.69251

**Published:** 2024-09-12

**Authors:** Atokali Chophy, Sweety Gupta, Deepa Joseph, Swati Verma, Manoj Gupta

**Affiliations:** 1 Department of Radiation Oncology, All India Institute of Medical Sciences, Rishikesh, Rishikesh, IND

**Keywords:** adaptive radiotherapy, cone beam computed tomography, head and neck cancer, image-guided radiation therapy, lateral neck diameter

## Abstract

Intensity-modulated radiation therapy (IMRT) has brought about interest in adaptive radiotherapy (ART) due to its benefit of accurately prescribing doses to tumors and sparing normal critical organs. Critical dosimetric errors and geometrical misses can occur due to anatomical changes during radiotherapy.

In the present study, five patients with head and neck malignancies undergoing radiation therapy were assessed for changes in primary gross tumor volume (GTVp), nodal gross tumor volume (GTVn), and clinical target volume-high risk (CTV-HR) using weekly megavoltage-cone beam computed tomography (MV-CBCT) scans. All patients had a reduction in GTV and lateral neck diameter (LND). There were reductions in tumor volumes leading to re-planning in the 20th fraction. Daily CBCT can guide the decision on the need for adaptation in patients with tumor volume reduction and with volumes going outside the body.

## Introduction

Adaptation in radiation oncology, especially in head and neck malignancies, has been a topic of interest in the last few decades with the advent of intensity-modulated radiation therapy (IMRT) [1]. IMRT allows for high radiation doses to tumors while reducing the doses to surrounding structures; however, anatomical and geometric changes over the course of radiation therapy may limit the benefits [2].

Image-guided radiation therapy (IGRT) with cone beam computed tomography (CBCT), either kilovoltage-CBCT (kV-CBCT) or megavoltage-CBCT (MV-CBCT), and adaptive radiation therapy (ART) with mid-treatment computed tomography (CT) scans are strategies devised to account for geometric misses, improve coverage of target volumes, and spare the organs at risk (OARs) [3].

Weight loss and a reduction in lateral neck diameter (LND) have also been reported in patients during radiation treatment [4]. However, currently, no guidelines have been established regarding the timing for adaptive re-planning. Significant changes have been reported in the third to fourth week [5,6]; hence, for adaptive re-planning, the 20th fraction was chosen.

This case series highlights the use of daily MV-CBCT monitoring of treatment volumes, along with LND, to assess the need for adaptive re-planning.

## Case presentation

Methods

Patient Selection

A total of five patients with histologically proven squamous cell carcinoma of the head and neck were analyzed for this case series.

Simulation

Radiation treatment simulations were done on the in-house CT simulator. All patients were simulated with a 5-clamp thermoplastic cast. CT images, with a 2.5 mm slice thickness, were obtained from the vertex to the tracheal bifurcation, both before and after giving intravenous non-ionic contrast.

IMRT Planning

Planning was done in the Eclipse 16.1 planning system (Varian Medical Systems, Palo Alto, CA, USA). All patients were treated using the volumetric arc therapy (VMAT) technique, with the radiation dose, as per the physician’s choice, ranging from 66 to 69.96 Gy in 30 to 33 fractions at five fractions per week. The planning margins were 5 mm to the clinical target volume (CTV). The dose constraints for OARs were as per the Quantitative Analyses of Normal Tissue Effects in the Clinic (QUANTEC) guidelines. Concurrent chemotherapy was Inj. cisplatin at 40 mg/m².

Daily MV-CBCT was done for patients prior to treatment. Patients were treated on the Halcyon v3 linear accelerator (Varian Medical Systems). All patients underwent a mid-treatment planning re-scan at four weeks. Every week during treatment, the tumor volumes were manually re-contoured by the same physician, and the LNDs were measured in the MV-CBCT. The parotid glands, mandible, and spinal cord were evaluated, and the window level was changed whenever required.

In the CBCT, changes in volume in the primary gross tumor volume (GTVp), nodal gross tumor volume (GTVn), CTV-high risk (CTV-HR), and LND were analyzed.

For dose calculation, the dose received by the OARs before re-planning (previous fractions) and after re-planning for the remaining fractions was calculated, and the sum of the doses received was analyzed.

Results

Characteristics

Patient characteristics are summarized in Table 1. There were four males and one female. The primary site was the oropharynx in three patients and the larynx in two patients. Three patients had American Joint Committee on Cancer (AJCC) Stage IVA disease, and two patients had Stage III disease. All patients received weekly concurrent cisplatin with radiation.

**Table 1:** Baseline characteristics of patients BOT: Base of tongue

Case	Site	Staging	Group stage	Gender
Case 1	Supraglottis	T3N0M0	III	Male
Case 2	Right BOT	T2N2aM0	IVA	Male
Case 3	Left tonsillar fossa	T2N2cM0	IVA	Male
Case 4	Supraglottis	T2N1M0	III	Female
Case 5	Right BOT	T4aN1M0	IVA	Male

Volumetric Changes

**Figure 1:**
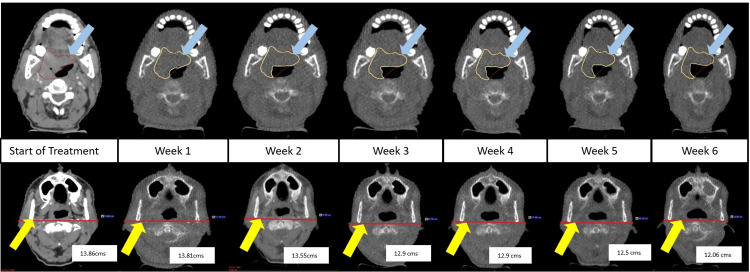
Weekly changes in the GTVp and LND in patient (Case 5) Blue arrows indicate GTVp, while yellow arrows represent LND. GTVp: Primary gross tumor volume; LND: Lateral neck diameter

**Figure 2:**
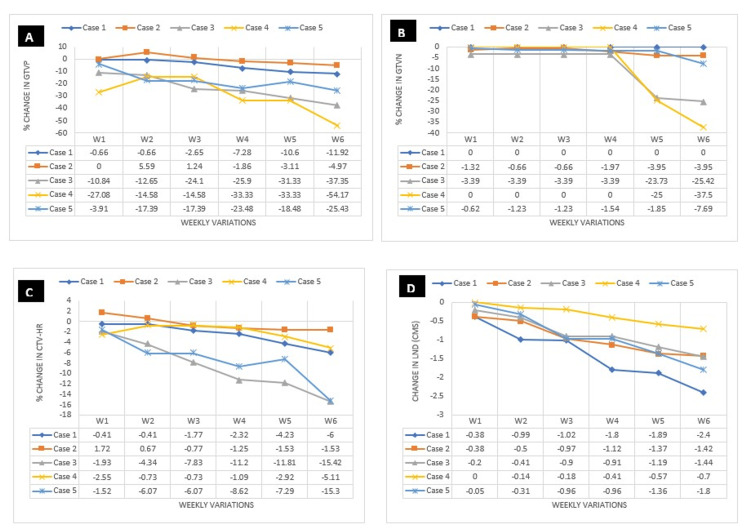
Trend line showing weekly volumetric variations A) Primary gross tumor volume (GTVp); B) Nodal gross tumor volume (GTVn); C) Clinical target volume-high risk (CTV-HR); D) Lateral neck diameter (LND) W1: Week 1; W2: Week 2; W3: Week 3; W4: Week 4; W5: Week 5; W6: Week 6; %: Percentage; CMS: Centimeters

Bone-to-bone matching was done, and mass changes in the target or OARs clearly visible on MV-CBCT were evaluated. There were changes in the volumes of GTVp, gradually from week 1 (W1) to week 6 (W6), with volumetric reductions ranging from -4.97% to -54.17% in the sixth week. Volumetric reductions were also noted in GTVn, ranging from -3.95% to -25.42%. In the HR-CTV, reductions in volumes were around -6% to -15.42%. LND changes were also noted, ranging from -0.7 to -2.4 cm (Figures 1-2).

Dosimetric Evaluation

Patients had undergone a mid-treatment re-planning in the fourth week (20th fraction), and the doses that the OARs and the tumor volumes received are summarized in Table 2.

**Table 2:** Dosimetric parameters of planning target volume (PTV) and organs at risk (OARs)

Parameters		Case 1	Case 2	Case 3	Case 4	Case 5
PTV V95%	Initial	99.07%	96.59%	97.44%	97.48%	97.09%
	Re-plan	98.98%	97.85%	99.53%	99.72%	98.95%
Right parotid (Dmean)	Initial	25.6 Gy	31.67 Gy	24.68 Gy	30.34 Gy	35.47 Gy
	Re-plan	27.97 Gy	32.77 Gy	25.20 Gy	42.02 Gy	38.53 Gy
Left parotid (Dmean)	Initial	30.36 Gy	33.17 Gy	45.11 Gy	25.89 Gy	33.56 Gy
	Re-plan	32.85 Gy	34.94 Gy	37.94 Gy	25.11 Gy	32.76 Gy
Brainstem (Dmax)	Initial	28.69 Gy	42.46 Gy	43.94 Gy	44.11 Gy	52.48 Gy
	Re-plan	30.85 Gy	41.52 Gy	44.92 Gy	43.40 Gy	45.28 Gy
Spinal cord (Dmax)	Initial	34.58 Gy	39.92 Gy	39.24 Gy	33.33 Gy	42.86 Gy
	Re-plan	35.86 Gy	34.94 Gy	38.65 Gy	34.29 Gy	39.01 Gy
Mandible (Dmax)	Initial	63.17 Gy	74.56 Gy	74.36 Gy	61.79 Gy	68.41 Gy
	Re-plan	63.13 Gy	74.37 Gy	74.10 Gy	63.81 Gy	68.94 Gy
Ipsilateral parotid (Dmean)	Initial	25.6 Gy	33.17 Gy	24.68 Gy	30.34 Gy	35.47 Gy
	Re-plan	27.97 Gy	34.94 Gy	25.20 Gy	42.02 Gy	38.53 Gy
Contralateral parotid (Dmean)	Initial	30.36 Gy	31.67 Gy	45.11 Gy	25.89 Gy	33.56 Gy
	Re-plan	32.85 Gy	32.77 Gy	37.94 Gy	25.11 Gy	32.76 Gy

Toxicities During Radiation

On weekly evaluation during radiation therapy, Grade 2 dermatitis was seen in one patient, while all others had Grade 1 dermatitis. All patients had Grade 1 mucositis, except for one patient who had Grade 2 mucositis. Grade 3 laryngitis was observed in a patient with a larynx primary.

Follow-Up

Four patients had a complete response on imaging three months after treatment. One patient had a residual neck node on imaging, which was positive for malignancy on fine needle aspiration. He underwent a neck nodal dissection, in which the neck nodes, on histopathology, were negative for malignancy. All patients, at two years post-treatment, are currently on follow-up with no evidence of disease.

## Discussion

The timing of re-scanning and re-planning of patients during radiation therapy is still a matter of debate. Barker et al. concluded that changes in target volumes and critical structures appeared to be significant in three to four weeks during treatment [7]. In our series, we have also seen significant changes in the GTVp and CTV-HR that occurred in the fourth week. However, for the GTVn, the changes are not clearly appreciable, unless the node is nearer to the body surface and is outside the body.

LND, or the lateral neck separation reduction, was seen in the range of -0.5 to -1.8 cm by Capelle et al. [8], which was higher in our case series, ranging from -0.7 to -2.4 cm. Reduction of LND leads to an increase in the incidence of the volume of the PTV going outside the body. Whether this has an effect on the potential benefit of IMRT is yet to be investigated.

Dose to the OARs is most commonly studied in ART. Our series observed an increase in the total actual mean dose received by the ipsilateral parotid in all five patients (0.52 to 11.68 Gy). For the contralateral parotid, two patients had an increase in the total actual mean dose received, while three patients had a reduction in dose (-0.7 to -7.17 Gy). Studies have reported a reduction in mean parotid doses from -0.6 to 4.1 Gy [9].

Spinal cord maximum dose variations were increased in two patients (0.96 to 1.28 Gy) and reduced in three patients (0.59 to 4.98 Gy). The median dose reduction was reported as 6 Gy [8,10].

Mandible Dmax was also increased in two patients (0.53 to 2.02 Gy) and decreased in three patients (-0.04 to -0.26 Gy). Dosimetric improvements can be achieved by one properly timed re-plan, as concluded by Schwartz et al. [11].

A study by Chen et al. reported local control at 88% at two years for patients who underwent ART [12]. All five patients had excellent local control on follow-up at two years and are currently on follow-up in the case series.

The limitations were uncertainties in judging the change in nodal volumes unless they were going outside the target volumes or the body. Small OARs, such as the chiasma, optic nerves, lens, and cochlea, are difficult to evaluate on MV-CBCT.

## Conclusions

We have described a case series of five patients utilizing weekly MV-CBCT to assess the volumetric changes in the tumor volumes and LND. With currently no fixed guidelines for the timing of ART in the present scenario, daily CBCT can be a guide to deciding the need for adaptation in patients with tumor volume reduction and volumes going outside the body, utilizing existing facilities and adding no extra cost.
